# Is It About Hierarchy? An Evolutionary Account of the Burnout–Depression Overlap

**DOI:** 10.3390/bs16081289

**Published:** 2026-07-28

**Authors:** Hector A. Garcia, Jarad J. Reiss

**Affiliations:** 1 Department of Health and Behavioral Sciences, Texas A&M University-San Antonio, San Antonio, TX 78224, USA; reissj@uthscsa.edu; 2 Department of Psychiatry, University of Texas Health Science Center San Antonio, San Antonio, TX 78229, USA

**Keywords:** occupational burnout, depression, social rank, evolutionary psychology, workplace hierarchies

## Abstract

The distinction between occupational burnout and depression remains uncertain. This study examined whether burnout may represent a context-sensitive expression of depressive responses to social threat, particularly those related to subordination and social exclusion in workplace hierarchies. Drawing on the Social Competition and Social Risk Hypotheses, we assessed associations among burnout, depression, and theoretically relevant indicators of workplace hierarchy and social competition, including perceived social rank, leader dominance and prestige, negative workplace gossip, and perceived physical vulnerability. Participants completed self-report measures of burnout, depressive symptoms, and workplace social dynamics. Correlational and exploratory cluster analyses examined the overlap between burnout and depression. Burnout and depression were highly correlated and exhibited parallel associations with workplace hierarchy and social threat variables, including lower perceived social rank, greater exposure to negative workplace gossip, and higher perceived physical vulnerability. Exploratory cluster analysis identified two profiles characterized by relatively low versus high burnout and depression, consistent with a shared underlying pattern of responding. Burnout showed somewhat stronger associations with leader dominance and prestige than depression, suggesting greater sensitivity to workplace hierarchy. These findings are consistent with the possibility that burnout may represent a context-sensitive expression of depressive processes and have implications for diagnostic classification, intervention strategies, and theoretical integration across occupational, clinical, and evolutionary perspectives.

## 1. Is It About Hierarchy? An Evolutionary Account of the Burnout–Depression Overlap

While burnout has become a popular construct, its definition remains contested. Burnout is variously described as a work-related stress reaction, a psychological syndrome, or a form of depression (Bianchi & Schonfeld, 2024; Lastovkova et al., 2018; World Health Organization, 2022). This lack of consensus has hindered the development of theoretically grounded models to guide assessment, prevention, and intervention.

The Maslach Burnout Inventory (MBI; Maslach & Jackson, 1981) is the most widely used tool for assessing burnout. The MBI approaches burnout as a three-component syndrome comprising: exhaustion (feeling depleted), cynicism (detachment toward work and others), and reduced professional efficacy (overly negative appraisal of one’s performance). The MBI is meant to be used for quantifying burnout symptoms and does not allow diagnoses to be established; burnout is not recognized as a mental disorder in the DSM-5-TR (American Psychiatric Association, 2022), and the ICD-11 defines it as an occupational phenomenon, rather than a medical condition (World Health Organization, 2022). Its diagnostic ambiguity may partly stem from its overlap with depression.

A growing body of research suggests burnout reflects a depressive condition. Studies have found high correlations (Valente et al., 2018) and suggest that they represent the same factor (Bianchi et al., 2021). Longitudinal evidence indicates that burnout and depression often exacerbate and remit in tandem (Ahola et al., 2014), and psychometric research has found that items on burnout measures correlate more strongly with depressive measures than with the burnout measures themselves (Bianchi et al., 2021). Sen (2022) concluded that depression and burnout are predicted by essentially the same (work and nonwork) factors. Research on cognitive functioning found that burnout is associated with attentional, memory, and interpretation biases that are typical of depression (e.g., Bianchi et al., 2020). Moreover, both depression and burnout involve similar feelings of impotence—burnout has been described as when workers feel “helpless, hopeless, and powerless” (Schaufeli & Enzmann, 1998, p. 25), whereas helplessness and powerlessness are central depressive symptomatology (Pryce et al., 2011; Willner et al., 2013). Such findings suggest that burnout may reflect an expression of broader depressive processes.

Symptomatic and conceptual overlap between burnout and depression, along with ongoing definitional debate, highlight the need for theoretical models that move beyond symptom comparisons. Evolutionary models of depression offer a framework for understanding shared etiology, proposing that depressive responses may have evolved to manage ancestral pressures such as social competition and exclusion—pressures that continue to shape stress responses in the modern workplace. Emerging scholarship suggests that mismatches between contemporary work environments and our evolutionary past—where social competition and exclusion often carried physical risks—may underlie that stress and contribute to burnout (van Vugt et al., 2024).

According to the Social Competition Hypothesis (Price et al., 1994), depressive symptoms have been proposed to reflect an evolved strategy for managing dangerous social competition. This perspective posits that depression functions as an appeasement strategy to avert conflict with higher-ranking individuals, de-escalate aggression, and promote acceptance of social defeat. Specific symptoms, such as anhedonia—e.g., the loss interest in food or sex—may serve to diminish motivation to compete for resources central to evolutionary fitness (Sloman et al., 2003). Self-esteem—conceptually related to the Professional Efficacy dimension of the MBI—has been seen as a social rank gauge guiding individuals to either challenge or submit based on their relative position of power (Mahadevan et al., 2016).

Similarly, the Social Risk Hypothesis (Allen & Badcock, 2003) posits that depression functions as an appeasement response to perceived threats of social exclusion, serving to reduce social threat and enhance reintegration. These models are supported by findings that low perceived social rank is associated with depressive symptoms (Wetherall et al., 2019) and that individuals with depression show increased sensitivity to social threat cues (Leppänen, 2006) and tend to render more accurate social appraisals (Moore & Fresco, 2012).

Arguments distinguishing burnout from depression often emphasize burnout’s greater expression in the workplace (e.g., Maslach et al., 2001). Differences in expression, however, may be related to the fact that modern workplaces are often highly ranked-stratified, with formal hierarchies, performance monitoring (where individuals are evaluated and ranked), and resource control that make status both visible and consequential (Garcia, 2024). Thus, the modern workplace may uniquely elicit appeasement responses, especially under dominance-based leadership, low perceived status, or negative peer signaling.

Although modern workplaces rarely involve physical danger, cues of subordinate social status or social exclusion may nevertheless be associated with perceptions of physical vulnerability because they engage psychological mechanisms shaped by ancestral environments in which status competition frequently carried substantial physical risk. Garcia et al. (2025) found that domineering leadership, low rank, and other competition-related variables—such as perceived physical vulnerability—predicted burnout. Likewise, negative workplace gossip has been linked to burnout (Georganta et al., 2014) and may signal coalitionary threat (Garcia, 2024). Recent work further suggests that modern workplace cues such as negative gossip and hypergaze may activate these same evolved threat-detection mechanisms, providing additional support for viewing burnout through the lens of social competition and coalitionary threat (Garcia et al., 2026). Although workplace aggression, poor leadership, and adverse social interactions have long been associated with burnout (Gascón et al., 2013; Maslach & Leiter, 2016; Savicki et al., 2003), the present framework suggests that these seemingly distinct workplace experiences may represent different responses to rank competition and social exclusion.

Given the substantial empirical overlap between burnout and depression, and theoretical support for shared evolutionary origins, the possibility that burnout may reflect context-specific appeasement behavior consistent with evolutionary models of depression warrants investigation.

The present study therefore examined correlations between burnout, depression, and theoretically relevant indicators of workplace hierarchy and social competition, including perceived social rank, leader dominance and prestige, negative workplace gossip, and perceived physical vulnerability. If burnout and depression are similarly associated with these variables, they may represent context-specific expressions of a common underlying psychological system. Although the present study cannot directly test evolutionary mechanisms, it can evaluate whether the observed pattern of associations is consistent with predictions derived from the Social Competition and Social Risk Hypotheses.

### Current Research

The present study examined whether burnout and depression similarly relate to indicators of rank and social threat in the workplace. Drawing from the Social Competition (Price et al., 1994) and Social Risk (Allen & Badcock, 2003) Hypotheses, we expected that:

1. Burnout and depression would be strongly positively correlated.
2. Burnout and depression would exhibit similar patterns of association with rank and competition variables such as leader dominance, coalitionary threat (e.g., workplace gossip), physical vulnerability and perceived rank.

Exploratory cluster analyses were conducted to further would emerge characterized by shared burnout–depression profiles and distinct social-cognitive patterns relating to hierarchy, power, and threat.

## 2. Method

This cross-sectional observational study followed procedures consistent with Garcia et al. (2025). The Institutional Review Board at Texas A&M University–San Antonio (Protocol #2024-28) approved the study. The project was preregistered on the Open Science Framework (OSF; https://osf.io/bypc8) prior to conducting research, and that registration adheres to the disclosure requirements of the institutional registry. Additional methodological details and study materials are available through the preregistration. A G*Power version 3.1.9.7 analysis estimated that 134 participants were needed to detect a small to moderate effect size (*f*^2^ = 0.10, α = 0.05, β = 0.80). A total of 316 participants were recruited through Prolific and were compensated $16 USD per hour for their participation. The final sample after exclusions consisted of 256 participants. Participants were required to be at least 18 years of age, currently employed, and proficient in English. Participants were excluded for failing embedded attention checks, incomplete survey responses, duplicate participation, or failing to meet prespecified eligibility criteria described in the preregistration.

Participants ranged from 18 to 66 years (*M* = 46, *SD* = 12.3), with 50.6% reporting as female. Most participants identified as White (71.0%), followed by Black (10.8%), Hispanic (7.3%), Asian (6.9%), Other (2.3%), American Indian or Alaska Native (1.2%), and Middle Eastern or North African (0.4%). Overall, 37.5% of participants reported no supervisory role, 10.9% identified themselves as first-line supervisors, 19.5% as team leaders, 24.6% as managers, 6.3% as executives, and 1.2% as senior executives.

### 2.1. Materials

Participants completed an online survey which included self-report measures assessing social rank perceptions, organizational status/supervisory role, burnout, depression, negative workplace gossip, perceived physical vulnerability, and leadership dominance and prestige. The survey also included demographic questions and embedded attention checks. Demographic questions were presented at the end of the survey to minimize potential priming effects.

### 2.2. Measures

#### 2.2.1. Burnout

Burnout was measured using the Maslach Burnout Inventory—General Survey (MBI-GS; Schaufeli et al., 1996), a 16-item self-report instrument created to assess three dimensions of burnout: exhaustion, cynicism, and professional efficacy. Participants responded using a six-point Likert scale ranging from Never to Daily. Cronbach’s alpha was 0.90.

Consistent with prior studies using composite MBI scores (e.g., Braun et al., 2024; Rila et al., 2025), and evidence that a dominant general burnout factor accounts for most of the variance across MBI items (Aguayo-Estremera et al., 2024), analyses focused on an overall burnout score calculated by averaging the three MBI subscale scores. This approach was consistent with the present study’s aim of examining burnout as a unitary construct in relation to depression.

#### 2.2.2. Depression

Depression was assessed using the PHQ-8 (Kroenke et al., 2001), assessing symptoms over the past two weeks on a four-point Likert scale ranging from 0 (Not at all) to 3 (Nearly every day). Total scores were calculated by summing items responses, yielding a possible range of 0 to 24, with higher scores indicating greater depressive symptom severity. Scores of 10 or greater are commonly used as the cutoff for probably major depression, where scores of 20 or higher indicate severe depressive symptoms. Example items from the scale included statements such as, “Little interest or pleasure in doing things.” Cronbach’s alpha had a value of 0.90.

#### 2.2.3. Negative Gossip

Negative workplace gossip was assessed using a three-item scale created by Chandra and Robinson (2009). Participants rated their agreement to statements such as “In the past six months, others spread rumors about me in the workplace” on a five-point Likert scale ranging from 1 (Never) to 5 (Daily). Responses were summed to create a total score, with possible scores ranging from 3 to 15. Higher scores indicated greater exposure to negative workplace gossip. Cronbach’s alpha reached 0.91.

#### 2.2.4. Social Rank

Perceptions of social rank were assessed by the Social Comparison Scale (SCS), an 11-item measure prompting participants to select how they would describe themselves in comparison to others on a 10-point scale, for example, “Untalented” (1 = *Untalented*, 10 = *More Talented*; Allan & Gilbert, 1995). Item scores were summed to produce a total score ranging from 11 to 110, with higher scores indicating greater perceived social rank relative to others. Cronbach’s alpha was 0.90.

#### 2.2.5. Dominance

Leader dominance was measured using the dominance subscale from the Dominance and Prestige Scale (Cheng et al., 2010). Participants rated their immediate supervisor. This subscale included eight items, rated on a seven-point Likert scale ranging from 1 (Not at all) to 7 (Very much). Item scores were summed to produce a total score ranging from 8 to 56, with higher scores indicating greater perceived leader dominance. Example items include statements such as “I enjoy having control over others.” The internal reliability of the scale in the current study was Cronbach’s alpha was 0.90.

#### 2.2.6. Prestige

Leader prestige was measured using the prestige subscale from the Dominance and Prestige Scale (Cheng et al., 2010). Participants rated their immediate supervisor. This subscale included eight items, rated on a seven-point Likert scale ranging from 1 (Not at all) to 7 (Very much). Item scores were summed to produce a total score ranging from 8 to 56, with higher scores indicating greater perceived leader prestige. Example items include statements such as “He/she is held in high esteem by members of the group.” The internal reliability of the scale in this study was Cronbach’s alpha was 0.93.

#### 2.2.7. Physical Vulnerability

Perceptions of physical vulnerability were measured using the Physical Vulnerability subscale of the Likelihood of Experiencing Physical and Nonphysical Events in the Future Questionnaire (Dean et al., 2019). This subscale assessed the perceived likelihood of experiencing physical harm and consisted of 14 items rated on a five-point Likert scale ranging from 1 (Extremely unlikely) to 5 (Extremely likely). Item scores were summed to produce a total score ranging from 14 to 70, with higher scores indicating greater perceived physical vulnerability. The internal reliability of the scale was Cronbach’s alpha was 0.93.

#### 2.2.8. Organizational Status

Organizational status was assessed using a single item developed for the present study. Participants selected one of six predefined categories reflecting increasing formal authority: “(1) *None*, (2) *Team Leader* (informal, not responsible for rating performance), (3) *First-line Supervisor* (formal, responsible for rating performance), (4) *Manager* (e.g., division, service, etc.), (5) *Executive* (e.g., associate director, chief of staff, program director), and (6) *Senior Executive* (e.g., President, Network Director, CEO)”. Responses were coded from 1 to 6, with higher scores indicating greater formal organizational status.

### 2.3. Procedure

The procedure followed Garcia et al. (2025). After providing informed consent, eligible participants completed an online Qualtrics survey administered through Prolific. Embedded attention checks were included throughout the survey. Upon completion, participants received debriefing information and mental health resources related to workplace burnout.

### 2.4. Data Analysis

We first examined the associations among burnout, depression, and our focal variables using both Pearson (parametric) and Spearman (nonparametric) correlation analyses. This allowed us to capture both linear and monotonic relationships.

We also conducted an exploratory cluster analysis to complement the correlational analyses by examining whether participants naturally organized into burnout–depression profiles. A profile-based approach therefore provides an additional method for examining whether burnout and depression tend to co-occur within individuals or instead organize into distinct symptom configurations.

To examine this, a two-step cluster analysis was then conducted as an exploratory approach to identifying naturally occurring profiles within the sample based on participants’ burnout and depression scores, both treated as continuous variables. Prior to analysis, the two variables were standardized (*z*-scored) to place them on a common scale, facilitating score comparisons. The two-step procedure begins by pre-clustering the data into small sub-clusters, which are then grouped into final clusters using a hierarchical method. The optimal number of clusters was determined automatically (i.e., no number was prespecified) using the Bayesian Information Criterion (BIC), and cluster quality was evaluated using the silhouette coefficient. We employed cluster analysis to examine whether burnout and depression symptoms aligned into convergent profiles—for example, whether higher burnout co-occurred with higher depression within individuals.

To assess differences across the identified clusters, we compared them on all focal variables using Welch’s analysis of variance (ANOVA). Welch’s ANOVA is robust to violations of the homogeneity of variance assumption and to unequal sample sizes—conditions commonly encountered in cluster-derived groups. Separate Welch’s ANOVAs were used because the analyses focused on individual theoretically relevant outcomes rather than a multivariate omnibus comparison. To quantify the magnitude of between-cluster differences, we computed Cohen’s *d*. Effect sizes were interpreted using Cohen’s (1988) conventional benchmarks, where *d* values of approximately 0.20, 0.50, and 0.80 are considered small, medium, and large, respectively.

## 3. Results

Descriptive statistics are available in Table 1. Burnout and depression correlated highly with one another, Pearson’s *r* = 0.60, *p* < 0.001, and Spearman’s *ρ* = 0.64, *p* < 0.001. On average, burnout symptoms did not correlate more highly with each other (mean *r* = 0.40; mean *ρ* = 0.46) than with depressive symptoms (mean *r* = 0.45; mean *ρ* = 0.48).

Burnout and depression exhibited comparable patterns of association with our focal variables (Table 2). Correlations with leader and prestige were, however, somewhat higher for burnout than for depression. Overall, correlations ranged from small (e.g., for organizational status) to nearly large (e.g., for social rank). All correlations were in the expected direction.

**Figure 1:**
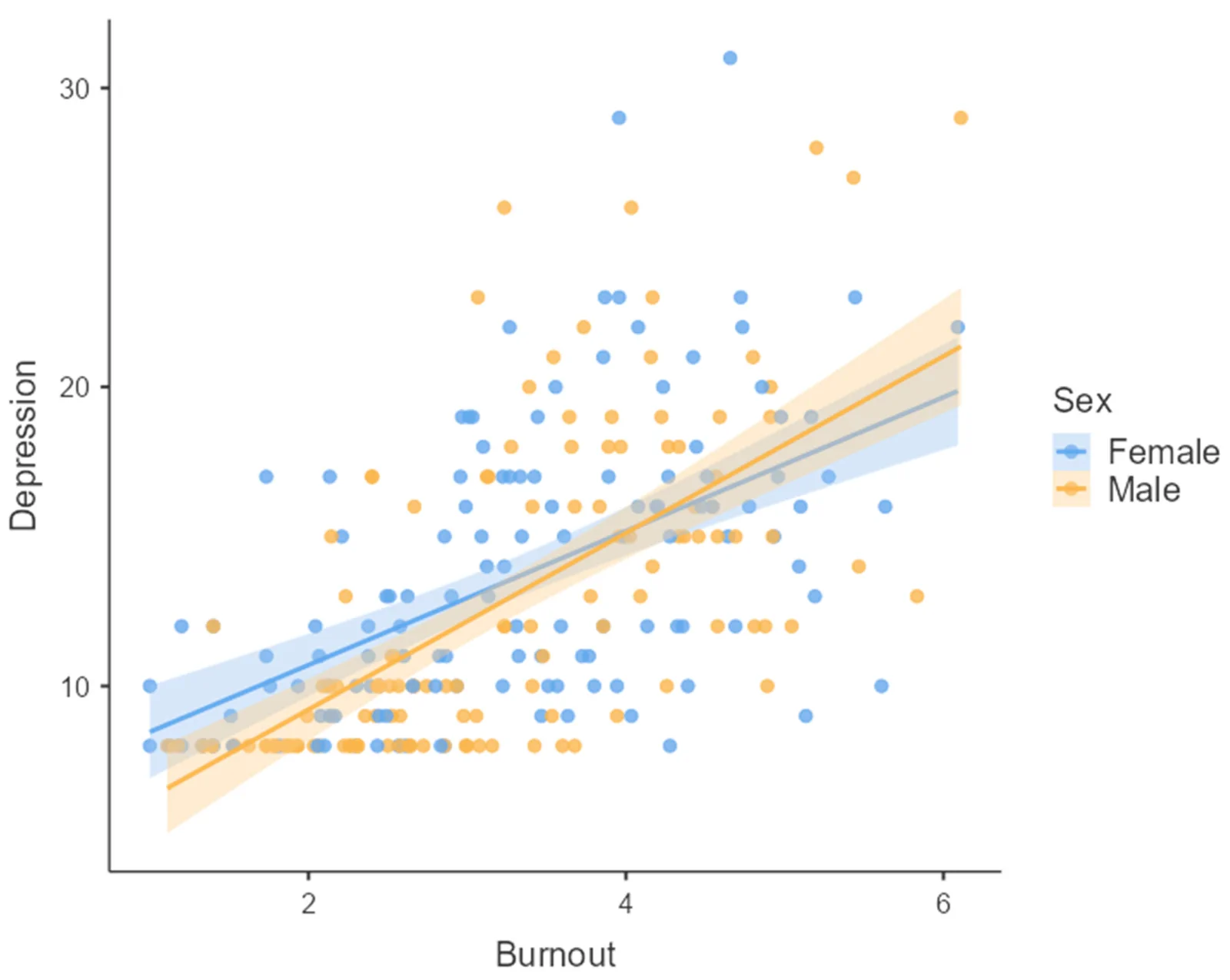
Burnout–depression association by sex. Exploratory cluster solution for burnout and depressive symptoms.

A two-cluster solution emerged from our cluster analysis (Table 3, Figure 1), with burnout and depression varying in close parallel. Cluster 1 comprised individuals with lower levels of burnout and depression, whereas Cluster 2 comprised individuals with higher levels of burnout and depression. The silhouette coefficient was 0.60, suggesting good cluster quality. Welch’s ANOVA revealed that the two clusters differed on all focal variables—social rank, organizational status, leader dominance, leader prestige, negative workplace gossip, and physical vulnerability. Effect sizes ranged from medium (e.g., organizational status) to large (e.g., social rank).

## 4. Discussion

The present findings are consistent with the hypothesis that burnout and depression may reflect expressions of a shared evolved response to social threat, particularly subordinated status and coalitionary exclusion. These findings offer new insight into the psychological mechanisms that may underlie distress in the modern workplace.

Our results showed high correlations between burnout and depression, replicating past research (Meier & Kim, 2022; Schonfeld et al., 2019), and provided novel evidence that both are linked to social rank and social exclusion. Burnout and depression showed comparable patterns of association with organizational rank, the presence of workplace gossip, and feelings of physical vulnerability. Perceived social rank showed one of the strongest associations with both burnout and depression, echoing prior work linking low rank perceptions with depressive symptoms (Price et al., 1994; Wetherall et al., 2019). Our results extend these findings by showing that social rank associations are not limited to trait depression, but apply equally to burnout.

Notably, burnout was more strongly correlated with perceived leader dominance and prestige than depression. One possible explanation is that the workplace is a modern environment in which social rank, evaluative threat, and leadership power are especially salient. Because the MBI explicitly assesses work-related symptoms, burnout may be more sensitive than depression to leadership variables indexing dominance and prestige. From this perspective, differences in how burnout and depression appear across contexts may reflect the flexible activation of a shared psychological response system. The impulse to withdraw or subordinate following status loss is inherently context-dependent and may therefore be especially evident in structured workplace hierarchies.

Our cluster analytic findings were consistent with our correlational results. Individuals grouped into two psychologically distinct profiles—low vs. high in both burnout and depression—that also differed in the expected direction on all social hierarchy variables. Importantly, the cluster solution revealed parallel elevations in burnout and depression, rather than distinct burnout-only or depression-only groups. This observed cluster pattern suggests that both constructs are context-specific expressions of a shared adaptive system that regulates responses to hierarchy, defeat, and coalitionary risk. Future studies using latent profile analysis or longitudinal designs could evaluate whether distinct burnout-specific profiles emerge.

Consistent with our cluster analyses, we found that burnout and depression increased in parallel for both sexes (see Figure 1). Sex differences were modest but suggest a slightly steeper association among men at higher burnout levels. This pattern is consistent with findings by Buunk et al. (2007) that men experience greater burnout in response to perceived status loss—because men’s reproductive success historically has more heavily depended on status, resource acquisition, and competitive success, modern work environments may activate status regulation mechanisms differently among men.

These findings may have implications for theory, diagnosis, and intervention. Current conceptual frameworks artificially separate burnout and depression based on context (work vs. non-work), despite evidence of shared symptomatology and underlying mechanisms. Viewing burnout as an *evolved contextual expression* of depressive processes offers a more parsimonious model that is both theoretically grounded and clinically useful.

If future research supports these findings, interventions may benefit from greater attention to status restoration, social reintegration, leadership practices, and workplace power dynamics in addition to stress reduction. Likewise, therapies that address sensitivity to social rank, interpersonal relationships, and workplace context may prove useful alongside existing approaches rather than treating burnout as an entirely separate syndrome.

This work bridges occupational health psychology, clinical science, and evolutionary theory. Taken together, the findings are consistent with a broader framework in which workplace distress—whether labeled burnout or depression—may reflect responses to perceived threats to social inclusion and rank that are shaped by evolved psychological processes and expressed according to context.

### Limitations and Future Directions

Several limitations warrant consideration. First, we relied on cross-sectional data, preventing causal inferences. While the associations identified are robust and theoretically grounded, longitudinal designs are needed to determine the temporal sequencing of status-related stressors and the emergence of burnout and depression. Further, all variables were assessed via self-report, introducing potential biases (e.g., social desirability bias), and common method variance may have inflated the observed associations. In addition, the present sample consisted solely of individuals working remotely as part of a larger study on telework and burnout. Our findings therefore may not extend to other populations or work designs. Further investigation is warranted to clarify how expressions of leader dominance shape responses across different work modalities, particularly in-person settings where status and social threat cues may be more salient. Finally, the cluster analysis was exploratory, and future studies should evaluate the robustness of these findings using alternative person-centered approaches, such as latent profile analysis. Our evolutionary framework is not intended to replace social or organizational explanations of burnout, but rather to complement them by providing a higher-order account of why workplace hierarchy and social exclusion may be psychologically consequential.

Future research should explore how individual differences—such as trait submissiveness and sensitivity to social rank—shape susceptibility to this shared burnout–depression phenomenon. Additionally, evaluating interventions that target hierarchical structure, leadership behavior, or social signaling may inform strategies for prevention and treatment.

## 5. Conclusions

The present findings are consistent with the possibility that burnout reflects a context-sensitive expression of depressive processes related to social threat and subordination. The similar pattern of associations observed for burnout and depression is consistent with shared underlying processes predicted by evolutionary models. Adopting an evolutionary framework may help clarify their overlap, bridge occupational and clinical science, and inform future research examining hierarchy, social exclusion, and distress in the workplace.

## Figures and Tables

**Table 1:** Descriptive Statistics for the Study Variables.

	Burnout	Depression	Social Rank	Organizational Rank	Leader Dominance	Leader Prestige	Negative Gossip	Physical Vulnerability
Mean	3.25	13.20	69.74	2.46	28.32	44.19	3.84	32.41
Median	3.22	12.00	69.50	2.00	27.00	46.00	3.00	34.00
Standard deviation	1.14	4.98	16.00	1.42	11.34	9.14	1.76	10.88
Minimum	1.00	8.00	15	1.00	9	8	3	14.00
Maximum	6.11	31.00	107	6.00	63	56	11	59.00
Skewness (*SE* = 0.15)	0.17	0.98	−0.07	0.45	0.60	−1.14	2.42	−0.11
Kurtosis (*SE* = 0.30)	−0.72	0.56	−0.01	−1.12	0.13	1.64	5.41	−0.74

*Notes. N* = 256. *SE* = standard error.

**Table 2:** Person and Spearman Correlations Between Burnout, Depression, and the Study Variables.

		Burnout	Depression
Social rank	Pearson’s *r*	−0.42	−0.46
	95% CI Upper	−0.31	−0.36
	95% CI Lower	−0.52	−0.55
	Spearman’s *ρ*	−0.41	−0.47
Organizational rank	Pearson’s *r*	−0.21	−0.19
	95% CI Upper	−0.09	−0.07
	95% CI Lower	−0.33	−0.31
	Spearman’s *ρ*	−0.22	−0.17
Leader Dominance	Pearson’s *r*	0.32	0.14
	95% CI Upper	0.42	0.26
	95% CI Lower	0.20	0.02
	Spearman’s *ρ*	0.28	0.14
Leader Prestige	Pearson’s *r*	−0.42	−0.20
	95% CI Upper	−0.32	−0.08
	95% CI Lower	−0.52	−0.31
	Spearman’s *ρ*	−0.45	−0.28
Negative gossip	Pearson’s *r*	0.33	0.37
	95% CI Upper	0.43	0.47
	95% CI Lower	0.22	0.26
	Spearman’s *ρ*	0.28	0.33
Physical vulnerability	Pearson’s *r*	0.33	0.36
	95% CI Upper	0.44	0.46
	95% CI Lower	0.22	0.25
	Spearman’s *ρ*	0.34	0.41

*Notes. N* = 256. All correlations are statistically significant at *p* < 0.05 or lower. CI = confidence interval.

**Table 3:** Comparisons of Study Variables Across the Two Identified Clusters.

	Cluster 1 (*n* = 151; 59%)		Cluster 2 (*n* = 105; 41%)		Welch’s ANOVA	Effect Size
	*M*	*SD*	*M*	*SD*	*p*-Value	Cohen’s *d*
Burnout *	−0.64	0.65	0.92	0.63	<0.001	2.43
Depression *	−0.63	0.48	0.90	0.86	<0.001	2.31
Social rank	74.95	14.74	62.24	14.78	<0.001	0.86
Organizational rank	2.72	1.49	2.10	1.22	<0.001	0.45
Leader Dominance	26.09	10.02	31.54	12.37	<0.001	0.49
Leader Prestige	46.35	8.50	41.08	9.18	<0.001	0.60
Negative gossip	3.31	0.82	4.60	2.38	<0.001	0.78
Physical vulnerability	29.74	11.07	36.24	9.40	<0.001	0.62

*Notes.* * *z*-scored. ANOVA = analysis of variance.

## Data Availability

The study was preregistered on the Open Science Framework (OSF) prior to data collection (https://osf.io/bypc8). Data are available upon request.
